# Carbon-ion scanning lung treatment planning with respiratory-gated phase-controlled rescanning: simulation study using 4-dimensional CT data

**DOI:** 10.1186/s13014-014-0238-y

**Published:** 2014-11-11

**Authors:** Wataru Takahashi, Shinichiro Mori, Mio Nakajima, Naoyoshi Yamamoto, Taku Inaniwa, Takuji Furukawa, Toshiyuki Shirai, Koji Noda, Keiichi Nakagawa, Tadashi Kamada

**Affiliations:** Research Center Hospital for Charged Particle Therapy, National Institute of Radiological Sciences, 4-9-1, Anagawa, Inage-ku, Chiba-shi, Chiba 263-8555 Japan; Department of Radiology, The University of Tokyo Hospital, 7-3-1, Hongo, Bunkyo-ku, Tokyo, 113-8655 Japan

**Keywords:** Carbon-ion beam, Four-dimensional, CT, Lung cancer, Respiratory motion, Scanning beam

## Abstract

**Background:**

To moving lung tumors, we applied a respiratory-gated strategy to carbon-ion pencil beam scanning with multiple phase-controlled rescanning (PCR). In this simulation study, we quantitatively evaluated dose distributions based on 4-dimensional CT (4DCT) treatment planning.

**Methods:**

Volumetric 4DCTs were acquired for 14 patients with lung tumors. Gross tumor volume, clinical target volume (CTV) and organs at risk (OARs) were delineated. Field-specific target volumes (FTVs) were calculated, and 48Gy(RBE) in a single fraction was prescribed to the FTVs delivered from four beam angles. The dose assessment metrics were quantified by changing the number of PCR and the results for the ungated and gated scenarios were then compared.

**Results:**

For the ungated strategy, the mean dose delivered to 95% of the volume of the CTV (CTV-D95) was in average 45.3 ± 0.9 Gy(RBE) even with a single rescanning (1 × PCR). Using 4 × PCR or more achieved adequate target coverage (CTV-D95 = 46.6 ± 0.3 Gy(RBE) for ungated 4 × PCR) and excellent dose homogeneity (homogeneity index =1.0 ± 0.2% for ungated 4 × PCR). Applying respiratory gating, percentage of lung receiving at least 20 Gy(RBE) (lung-V20) and heart maximal dose, averaged over all patients, significantly decreased by 12% (*p* < 0.05) and 13% (*p* < 0.05), respectively.

**Conclusions:**

Four or more PCR during PBS-CIRT improved dose conformation to moving lung tumors without gating. The use of a respiratory-gated strategy in combination with PCR reduced excessive doses to OARs.

## Background

Pencil-beam scanning carbon ion radiotherapy (PBS-CIRT) has been recently implemented in our institution and is being used for tumors not subject to respiratory motion, such as those in the pelvic and head and neck regions [1]. In contrast to the passive scattering beam irradiation technique, the PBS-CIRT does not require a compensating bolus or patient collimator. PBS-CIRT generally requires much less time to start treatment than passive beam therapy and is also useful for adaptive therapy such as re-planning during a course of treatment. Nevertheless, a major limitation of this method is the intra-/inter-fractional positional changes. The complicated interplay effect between the beam spot and tumor motion during treatment may result in dose degradation within the target and excessive doses to healthy adjacent tissues if positional changes are not taken into consideration.

One solution to the problem of delivering a prescribed dose to a moving target is phase-controlled rescanning (PCR) [2,3]. The effective use of PBS-CIRT for lung treatment requires an initial quantitative evaluation of the dose distribution by using lung 4-dimensional computed tomography (4DCT) data sets. In this study, we investigated the impact of PCR and intrafractional respiratory motion on lung dose distribution in PBS-CIRT and compared the results between ungated and gated strategies.

## Methods

### Patients

Fourteen patients with lung tumors treated by CIRT in our hospital were randomly selected. None were candidates for surgical resection due to medical reasons or patient refusal. All patients had stage I non-small cell lung cancer (NSCLC) or oligometastatic lung tumors as a single lesion. Patient characteristics are summarized in Table 1.

**Table 1:** **Patient characteristics**

**No.**	**Gender**	**Age (y)**	**T stage**	**GTV volume (cc)**	**Respiratory cycle (s)**	**Location**		**Position**	**Pathology**	**Tumor size (cm) (LR × AP × SI)**	**GTV-COM (mm) Ungate (LR × AP × SI)**	**GTV-COM (mm) Gate (LR × AP × SI)**
1	M	75	T2N0M0	5.8	4.1	Left upper lobe	S4	SP	ADC	3.4 × 3.2 × 1.7	1.0 × 1.1 × 1.4	0.2 × 0.2 × 0.4
2	M	84	meta	3.0	3.9	Right upper lobe	S3	SP	SCC	2.0 × 2.3 × 1.5	1.5 × 4.7 × 21.8	0.9 × 1.3 × 11.6
3	M	76	meta	7.2	3.8	Right lower lobe	S6	PR	meta	2.5 × 2.6 × 2.5	0.8 × 1.6 × 5.1	0.8 × 0.8 × 3.9
4	F	77	T2N0M0	4.0	3.3	Left upper lobe	S3	SP	SCC	2.5 × 2.8 × 1.4	1.4 × 1.7 × 5.8	0.5 × 0.8 × 2.1
5	F	58	meta	0.8	4.0	Right lower lobe	S7	SP	meta	1.3 × 1.2 × 1.1	0.8 × 3.1 × 11.9	0.8 × 0.8 × 3.4
6	M	65	T2N0M0	33.4	4.3	Right lower lobe	S9	PR	SCC	5.0 × 5.9 × 3.9	1.0 × 1.6 × 8.7	0.2 × 0.4 × 4.3
7	F	80	T2N0M0	13.1	4.2	Left upper lobe	S3	SP	ADC	3.7 × 3.7 × 3.2	2.0 × 2.6 × 6.9	0.5 × 0.3 × 3.6
8	M	75	T2N0M0	8.0	3.1	Right lower lobe	S10	PR	SCC	3.1 × 2.8 × 2.2	1.1 × 3.7 × 17.0	0.1 × 1.7 × 6.8
9	F	81	meta	0.9	2.7	Left upper lobe	S3	SP	meta	1.4 × 1.1 × 1.2	2.4 × 2.0 × 3.9	0.5 × 0.4 × 0.6
10	M	64	T2N1M0	31.1	4.4	Left upper lobe	S4	SP	SCC	4.8 × 4.7 × 4.9	0.7 × 1.6 × 2.3	0.7 × 0.7 × 0.5
11	F	65	meta	10.8	5.5	Right lower lobe	S7	SP	meta	2.7 × 3.0 × 2.8	1.1 × 1.5 × 2.1	0.7 × 1.8 × 0.4
12	M	61	T1N0M0	5.0	3.6	Right lower lobe	S8	PR	ADC	2.7 × 2.5 × 1.7	1.4 × 1.1 × 1.3	0.0 × 0.8 × 0.4
13	M	80	T1N0M0	5.0	3.4	Right middle lobe	S5	SP	ADC	3.0 × 2.6 × 2.4	2.2 × 1.0 × 0.8	2.2 × 0.0 × 0.9
14	F	79	T1N0M0	2.2	2.8	Right lower lobe	S9	PR	ADC	2.0 × 2.0 × 1.2	2.5 × 3.1 × 11.4	1.4 × 1.7 × 3.5
	Average	72.9		9.3	3.8					2.9× 2.9 × 2.3	1.4 × 2.2 × 7.2	0.7 × 0.8 × 3.0
	S.D.	8.5		10.3	0.7					1.1 × 1.3 × 1.1	0.6 × 1.1 × 6.4	0.6 × 0.6 × 3.1

*Abbreviations*: *ADC* adenocarcinoma, *Met*a metastasis, *SD* standard deviation, *SCC* squamous cell carcinoma, *SP* supine, *PR* prone, *GTV* gross tumor volume, *COM* 3-dimensional displacement of center of mass.

GTV-COM was calculated at the peak 3D distance using 4DCT data sets for all respiratory phases (10 phases) and the 3 phases around peri-exhalation for the ungated and gated strategies, respectively.

### Image acquisition

After providing written informed consent, patients underwent 4DCT under free-breathing conditions. 4DCT data were acquired by using a 256 multi-slice CT, which can be obtained approximately 13 cm in a single rotation, therefore, a single respiratory cycle was obtained during 4DCT imaging [4]. All patients were fixed on the patient bed in the supine or prone position using an immobilization device. The respiratory phase was monitored using a respiratory sensing system consisting of a position-sensitive detector and an infrared-emitting light marker (Toyonaka Kenkyujo, Osaka, Japan). For the reconstruction conditions, the voxel size was 0.78 × 0.78 × 0.50 mm and the 4DCT data sets were subdivided into 10 phases (T00: peak inhalation; T50: peak exhalation).

### Target definition

Gross tumor volume (GTV) and organs-at-risk (OARs), including the normal bilateral lung (excluding the defined GTV), spinal cord, and heart were manually delineated on T50. These contours were transferred to the other phases using B-spline-based deformable image registration (DIR) [5]. Clinical target volumes (CTVs) were created by adding 10 mm margins to the GTVs in all directions, and then, they were manually modified considering the distance from the target to chest wall and ribs.

Since charged particle beams are highly sensitive to geometrical variations in water-equivalent path length (WEPL), intrafractional variations result in either overshooting or undershooting the target. To account for these WEPL changes in all motion phases, the field-specific target volume (FTV) was designed using 4DCT data sets [6]. To account for intrafractional WEPL variations at the distal and proximal sides along the same ray-line at respective phases, the FTV for each beam direction was constructed by selecting the maximum and minimum WEPL values. Two types of FTV were calculated, the first using CTVs around exhalation (T40-T60) (FTVgated) and the second using CTVs around a full respiratory cycle (T00-T90) (FTVungated).

### Treatment planning

4D treatment planning was performed using 4DCT data in respective respiratory phases. Using single uniform field optimization, the FTV was enclosed at minimum by the 95% isodose line for the patient’s dose prescription. In accordance with our routine lung treatment protocol, 48Gy(RBE) in a single fraction was delivered via four beam angles from the ipsilateral side of the tumor [7].

A reference dose distribution (planning dose) was calculated based on a FTV created spot map from the 4DCT data set. The spots were equally distributed over all phases in order to avoid the interplay effect. In addition, dose distributions in each of the respective phases were calculated using 4DCT data sets for PCR at respective beam spot positions by considering dose rate, energy change time, and respiratory cycles. The accumulated dose distribution was then calculated (treatment dose) by using DIR, which transforms the respective phase doses to the reference phase (T50) [5]. Our DIR accuracy averaged over all patients was 0.9 ± 1.4 mm, which was derived from the 4DCT data using the manual point matching method. The beam weight maps for the respective FTVs were optimized to obtain the uniform relative biological effectiveness (RBE) weighted absorbed dose, which was calculated by considering the non-linearity of RBE based on an experimental study [8]. The number of rescannings was varied from 1 to 10 [2,3]. The starting phase of irradiation was T00 in the ungated strategy and T40 in the gated strategy. All beam spots of a layer were designed to be irradiated *n* times repeatedly within a single gating window. After finishing one layer, the energy was changed to irradiate the next proximal layer during the next gating window. Scanning beam irradiation time was longer than that for treatment planning CT acquisition. Therefore, when the accumulated dose was calculated, we assumed that the respiratory data of respective patients were repeated during scanning beam irradiation.

In our institution, PBS-CIRT is delivered using a range shifter and 11 synchrotron energies resulting in what we term hybrid depth scanning [9]. Beam spot positions and beam weights were optimized for the FTV. Spot spacing was 2.0 mm laterally and 3.0 mm in the beam direction, and lateral scatter (80-20%) was approximately 5.0 mm. Control times for the range shifter and synchrotron energy changes were 420 ms and 150 ms, respectively. We set the scanning speeds in the superior-inferior and left-right directions to 100 mm/ms and 50 mm/ms, respectively, and the scan path was optimized to minimize the total path length.

The results were then compared for the ungated and gated strategies. We assumed that the respiratory pattern remained unchanged throughout the treatment course. Dose-volume histograms (DVH) for the CTV and OARs at peak exhalation were calculated for both plans. The minimum/maximum dose (Dmin/max) and the dose delivered to 95% of the volume (D95) of the CTV were calculated at the reference phase. To evaluate the dose homogeneity, a dose homogeneity index (HI) was defined as the standard deviation (SD) of the dose voxel values within the CTV at the reference phase to the prescribed dose. For all OARs, the mean, maximum, and minimum doses were evaluated. The derived dosimetric parameters were averaged over all patients and their SD was calculated. The Wilcoxon signed-rank test was used to examine the differences in dose assessment metrics. For multiple comparisons, the Steel-Dwass test was performed. A probability value of less than 0.05 was considered to be significant. All 4D dose distributions were analyzed using the Aqualyzer system [10].

## Results

**Figure 1:**
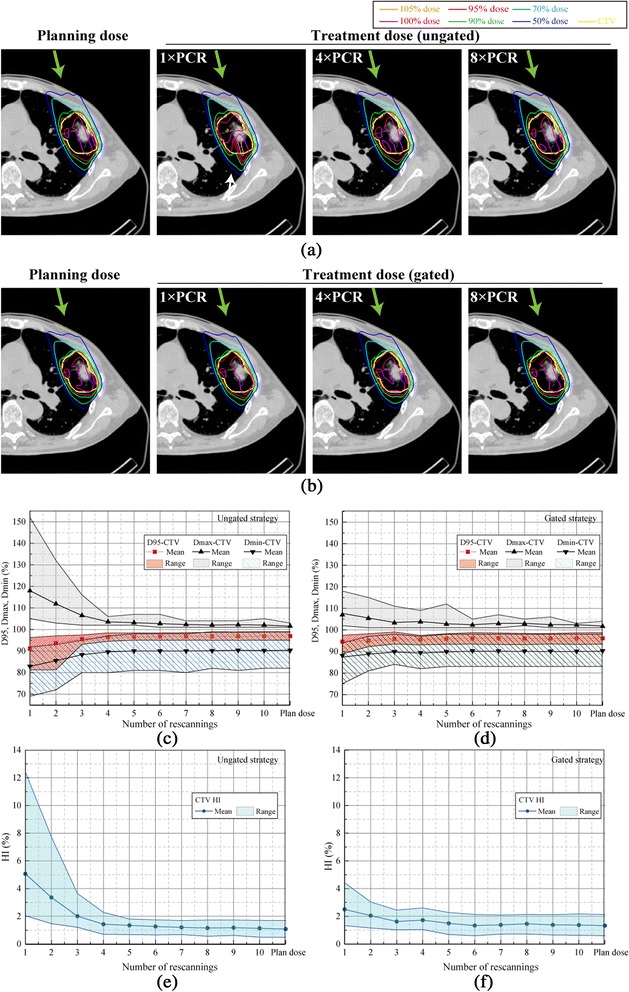
**Carbon-ion dose distributions with a single beam angle for (a) ungated and (b) gated irradiation.** Planning dose distribution and treatment dose simulations with 1 × PCR, 4 × PCR, and 8 × PCR. In the supine position, the beam angle was set to 340 degrees. The respiratory cycle was 4.2 sec. Yellow lines demonstrate the CTV (patient no. 7). Green arrows show beam direction. In Figure 1**(a)**, white arrow shows the dose degradation in the CTV. Dose assessment metrics for all 14 cases as a function of the number of rescannings. Beam angle was 340 degrees. D95, Dmax, and Dmin for **(c)** ungated and **(d)** gated strategies. The homogeneity index (HI) is for **(e)** ungated and **(f)** gated strategies.

The upper panels in Figure 1 give the accumulated dose distributions with a single beam angle when the number of rescannings was varied without respiratory gating (Figure 1a). The treatment dose differed from the planning dose distribution due to intrafractional motion. Target dose conformation improved with an increasing number of PCR. In a typical case, patient no. 7, for example, D95 values were 44.4 Gy(RBE), 46.7 Gy(RBE), and 46.7 Gy(RBE) with 1×, 4× and 8 × PCR, respectively. Furthermore, as shown in the lower panels of Figure 1b, the gating strategy achieved good dose conformation to the target even when the number of PCR was small; D95 with 1 × PCR in the gated plan (45.7 Gy(RBE)), for example, was better than that in the ungated plan (44.4 Gy(RBE)).

Dose assessment values averaged for all patients are summarized in Figure 1c-f. CTV coverage improved gradually as the number of rescannings increased in both the ungated and gated strategies. Although the gated strategy reduced the impact of the interplay effect by minimizing intrafractional tumor motion, four or more PCR were necessary to obtain the same results as the planning dose. Results are summarized in Table 2.

**Table 2:** **Dose assessment metrics averaged for all patients for 1 × PCR, 4 × PCR, and 8 × PCR**

**No. beam fields**	**Metrics**	**Strategy**	**Planning dose**		**1 × PCR**		**4 × PCR**		**8 × PCR**	
			**Mean ± (SD)**	**Range**	**Mean ± (SD)**	**Range**	**Mean ± (SD)**	**Range**	**Mean ± (SD)**	**Range**
1 beam field	D95 (Gy(RBE))	Ungated	46.5 ± 0.4	(45.6-47.4)	43.7 ± 1.7	(39.0-46.2)	46.3 ± 0.4	(45.4-47.2)	46.5 ± 0.4	(45.6-47.3)
		Gated	46.1 ± 0.6	(44.8-47.4)	45.4 ± 0.7	(42.7-46.8)	45.9 ± 0.5	(44.7-46.8)	46.1 ± 0.6	(44.7-47.2)
	Dmax (Gy(RBE))	Ungated	48.7 ± 0.3	(48.5-49.4)	56.6 ± 4.7	(50.4-73.0)	49.7 ± 0.6	(49.0-50.9)	49.0 ± 0.3	(48.5-49.9)
		Gated	48.9 ± 0.3	(48.5-49.4)	51.7 ± 1.9	(49.0-55.2)	49.9 ± 1.0	(48.5-53.8)	49.3 ± 0.5	(49.0-50.9)
	Dmin (Gy(RBE))	Ungated	43.4 ± 1.8	(39.4-47.5)	39.8 ± 3.0	(33.1-44.2)	43.0 ± 1.9	(38.4-46.6)	43.3 ± 1.8	(39.4-47.5)
		Gated	43.3 ± 1.4	(39.8-47.0)	42.0 ± 1.6	(36.0-45.1)	42.8 ± 1.3	(39.4-46.6)	43.2 ± 1.3	(39.8-47.0)
	HI (%)	Ungated	1.1 ± 0.2	(0.5-1.7)	5.1 ± 2.3	(2.0-12.4)	1.4 ± 0.3	(0.7-2.3)	1.2 ± 0.2	(0.6-1.7)
		Gated	1.3 ± 0.4	(0.6-2.1)	2.5 ± 0.8	(1.3-4.4)	1.7 ± 0.3	(1.0-2.6)	1.5 ± 0.4	(0.7-2.1)
	Treatment time (s)	Ungated	N/A	N/A	99.1 ± 50.7	(28.3-251.1)	99.1 ± 50.7	(28.3-251.1)	99.1 ± 50.7	(28.3-251.1)
		Gated	N/A	N/A	118.5 ± 55.0	(44.3-253.9)	118.8 ± 55.0	(47.1-269.8)	119.6 ± 55.5	(44.9-264.3)
4 beam fields	D95 (Gy(RBE))	Ungated	46.7 ± 0.4	(46.0-47.6)	45.3 ± 0.9	(43.0-46.3)	46.6 ± 0.3	(46.2-47.5)	46.7 ± 0.4	(46.0-47.6)
		Gated	46.3 ± 0.5	(45.5-47.6)	46.1 ± 0.4	(45.6-46.8)	46.2 ± 0.4	(45.6-47.2)	46.3 ± 0.6	(45.5-47.5)
	Dmax (Gy(RBE))	Ungated	48.5 ± 0.1	(48.5-49.0)	52.6 ± 2.2	(49.9-56.2)	48.9 ± 0.3	(48.5-49.4)	48.6 ± 0.2	(48.5-49.0)
		Gated	48.6 ± 0.2	(48.5-49.0)	49.8 ± 0.8	(49.0-50.9)	49.0 ± 0.5	(48.5-50.4)	48.8 ± 0.3	(48.5-49.4)
	Dmin (Gy(RBE))	Ungated	44.2 ± 1.7	(40.3-47.5)	42.7 ± 1.8	(39.4-45.1)	44.2 ± 1.7	(40.3-47.5)	44.2 ± 1.6	(40.8-47.5)
		Gated	44.4 ± 1.1	(42.7-47.5)	44.0 ± 0.7	(43.2-46.1)	44.2 ± 0.8	(43.2-46.6)	44.3 ± 1.1	(42.7-47.5)
	HI (%)	Ungated	0.9 ± 0.2	(0.3-1.2)	2.9 ± 1.1	(1.5-5.6))	1.0 ± 0.2	(0.4-1.2)	0.9 ± 0.2	(0.3-1.2)
		Gated	1.1 ± 0.3	(0.3-1.6)	1.5 ± 0.3	(1.1-2.0))	1.2 ± 0.2	(0.7-1.6)	1.2 ± 0.3	(0.4-1.7)
	Treatment time (s)	Ungated	N/A	N/A	396.4 ± 195.4	(180.7-915.8)	396.4 ± 195.4	(180.7-915.8)	396.4 ± 195.4	(180.7-915.8)
		Gated	N/A	N/A	474.1 ± 218.6	(235.1-939.1)	475.1 ± 218.7	(234.5-960.6)	478.5 ± 220.2	(239.2-956.7)

*Abbreviations*: *CTV* clinical target volume, *HI* homogeneity index, *PCR* phase-controlled rescanning, *SD* standard deviation.

**Figure 2:**
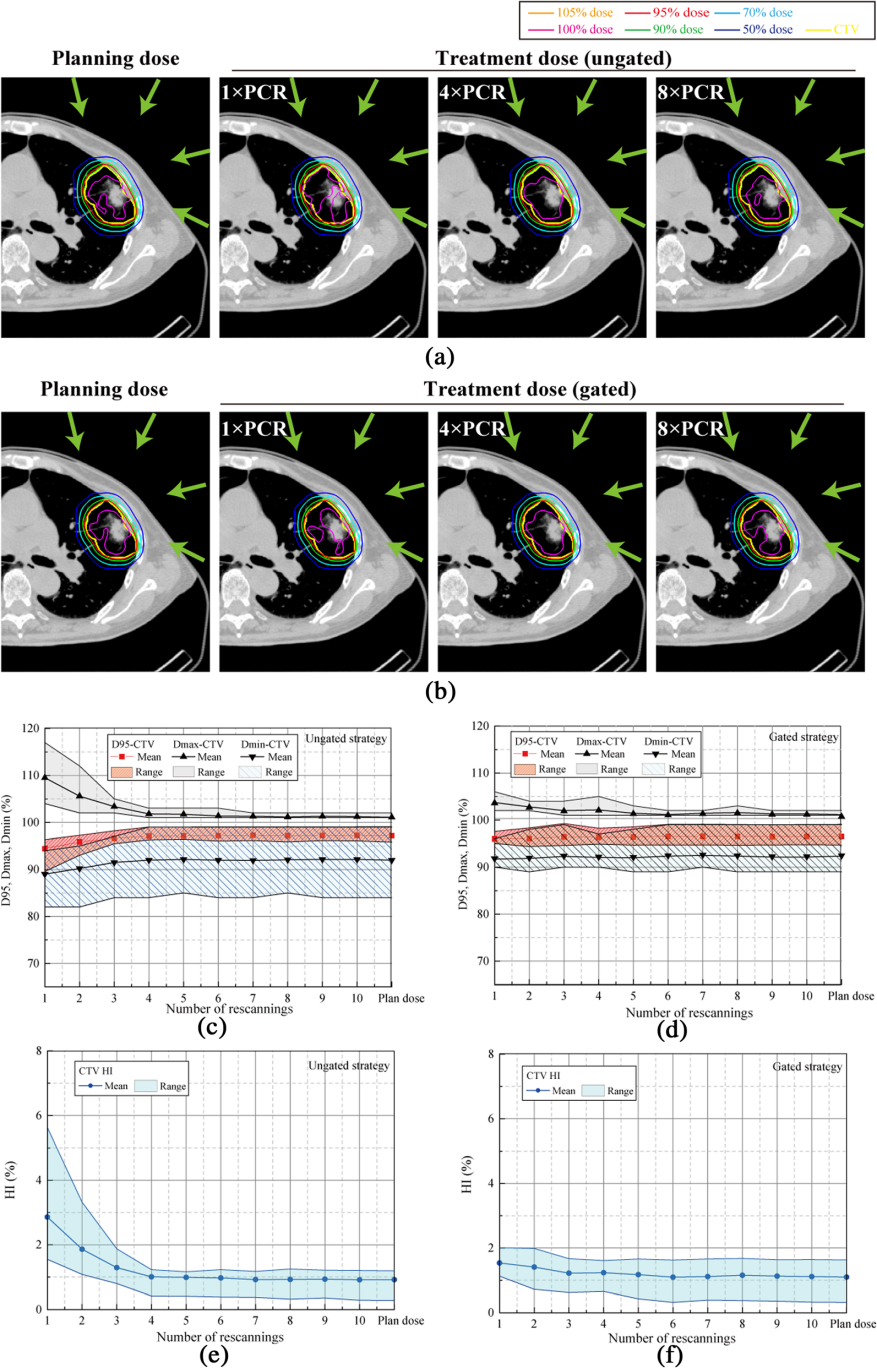
**Carbon-ion dose distributions with four beam angles for (a) ungated and (b) gated irradiation.** Planning dose distribution and accumulated dose simulations with 1 × PCR, 4 × PCR, and 8 × PCR. In the supine position, the beam angle was set to 20, 70, 110, and 340 degrees. The respiratory cycle was 4.2 sec. Yellow lines demonstrate the CTV (patient no. 7). Green arrows show beam direction. Dose assessment metrics for all 14 cases as a function of the number of rescannings. D95, Dmax, and Dmin for **(c)** ungated and **(d)** gated strategies. The homogeneity index (HI) is for **(e)** ungated and **(f)** gated strategies.

Figure 2 gives the dose distributions of a four-field plan for the same case. Without gating and a low number of PCR (e.g. 1 × PCR), the use of multiple coplanar fields reduced hot and cold spots and provided better conformation to the CTV than the single field mentioned above. D95, Dmax and Dmin values of the CTV improved with the use of multiple fields, from 44.4 Gy(RBE), 53.3 Gy(RBE), and 43.7 Gy(RBE) with a single field to 46.2 Gy(RBE), 49.9 Gy(RBE), and 45.1 Gy(RBE) with four fields, respectively. Moreover, the addition of gating provided better dose volume indices for the CTV than with the ungated plans. This tendency was particularly obvious with a single port with 1 × PCR (Figure 1), but the magnitude of improvement in dose uniformity became less prominent with 4× and 8 × PCR (Figure 1) and with four beam angles (Figure 2). With four beam fields using the gated strategy, an increase in PCR frequency provided only a slight improvement in dose metrics, with D95 of the CTV being 46.3 Gy(RBE), 46.4 Gy(RBE), and 46.4 Gy(RBE) for 1×, 4× and 8 × PCR, respectively.

Dose assessment values averaged for all patients for the four-field treatment are summarized in Figure 2c-f. In particular, four or more PCR resulted in adequate target coverage (CTV-D95: 45.3 ± 0.9 Gy(RBE) for 1 × PCR; 46.6 ± 0.3 Gy(RBE) for 4 × PCR; and 46.7 ± 0.4 Gy(RBE) for 8 × PCR in the ungated strategy) and a better HI (2.9 ± 1.1% for 1 × PCR; 1.0 ± 0.2% for 4 × PCR; and 0.9 ± 0.2% for 8 × PCR in the ungated strategy). As shown in Table 2, as for D95 for 4 × PCR/8 × PCR, applying the gated strategy induced a slight decrease but its extent was small as a clinical acceptable level (>96% of the prescribed 48 Gy(RBE)). In contrast, the dose coverage for 1 × PCR was significantly lower than for 4× and 8 × PCR (CTV-D95, Dmax, and HI; Steel-Dwass test, all *p* <0.01) in the ungated strategy. 1 × PCR in the ungated plan was not enough to sufficiently reduce dose inhomogeneity, and Dmax/Dmin values for all patients reached 52.6 Gy(RBE)/42.7 Gy(RBE). With regard to OARs, significant sparing of healthy tissues was achieved under all the modes studied. Of particular note is that the gated strategy decreased the dose to normal surrounding tissues. The mean percentage of lung receiving at least 20 Gy(RBE) (lung-V20) value and heart Dmax with 1 × PCR significantly decreased by 12% (from 4.3% in the ungated strategy to 3.8% in the gated strategy, *p* <0.001) and 13% (from 7.8 Gy(RBE) in the ungated strategy to 6.8 Gy(RBE) in the gated strategy, *p* = 0.01), respectively. Further, lung-V20, and heart and spinal cord Dmax indices did not change with an increase in the number of PCR.

With the PCR method, the total time of dose delivery is basically the same because each layer is irradiated during a single gate window (Table 2). However, total irradiation times with gated plans are necessarily prolonged.

## Discussion

In this study, we evaluated 4D dose distributions with changes in the number of PCR (1 to 10 times) using 4DCT datasets for 14 patients with lung tumors, and compared results between ungated and gated strategies. Although several groups have introduced rescanning and gating strategies [11,12], specifications of their beam delivery systems and rescanning methods (layered or volumetric etc.) differed from those of the presented study. In order to simulate lung PBS-CIRT delivery, we calculated FTVs that take into account intrafractional range variations using 4DCT data for the dose prescription. For the 4D treatment planning, dose distribution was calculated as a function of each respiratory phase, and the accumulated dose was calculated by applying DIR. From this we determined that four or more PCR would provide acceptable dose conformity, independent of patient condition (tumor location/size and respiratory cycle, etc.). This simple approach can be easily integrated into clinical applications.

Knopf et al. reported that using multiple scanning beam directions improved dose homogeneity in three patients with liver conditions [11]. As shown in Table 2, improved dose coverage for the CTV (D95 ≥ 43.0 Gy(RBE) for all patients) was achieved even though neither gating nor rescanning were applied because four field plans were used and the averaging of multiple beam angles (Figures 1 and 2) improved dose conformation. Thus, multiple beam directions will be used in the scanning beam lung treatment based on our lung treatment protocol used in the passive beam treatment [7].

Kang et al. used an average CT approach to the moving target and succeeded in delivering the beam to lung tumors using a commercially available treatment planning system [13]. As their methods used a smeared beam field and virtual target density estimation within the target, intrafractional range variations were not fully considered. In contrast, we used the T50-4DCT image as a reference, and the respiratory gating window was set to peri-exhalation because the peak exhalation is the most stable and reproducible respiratory phase.

Based on these FTV settings, we then analyzed the effectiveness of increasing PCR frequency. With an adequate number of rescannings, PCR averaged out the interplay effect between beam spot and intrafractional motion. Four or more PCR improved dose homogeneity to the moving target. This study therefore confirmed our previous observations in a numerical phantom [3].

Further more than 4 × PCR did not substantially improve target coverage and homogeneity as can be seen from Figures (Figures 1c-f, 2c-f). This plateau can be explained as follows; 4 × PCR provides adequate averaging out of the interplay effect and further rescanning provides no additional improvement in dose conformation. Furthermore, beyond the number of PCR, the additional averaging effect improves dose conformation via several other mechanisms. During PCR to one layer, the scan trajectory inverts after each single scan of an iso-energy layer. Further, during the peak expiratory phase (T50) the direction of respiratory tumor motion is reversed due to the switch from the inspiratory to expiratory phase.

The effect of an increase in PCR on dose metrics was larger for the ungated than for the gated strategy because gating itself causes an improvement in dose homogeneity by minimizing the amplitude of motion (Figures 1e,f, 2e and f).

For all CTV metrics listed in Table 2, the gated strategy was clearly superior to the ungated strategy with 1 × PCR. This does not mean that the gating method can replace the benefits of PCR. Residual intrafractional motion exists within the shrunken FTV, and the interplay effect between the beam spot and tumor motion during the gating window leads to dose inhomogeneity with 1 × PCR (much slow scanning speed), even with gating. This is emphasized with a single beam angle. The multiple PCR technique should therefore be used regardless of whether the ungated or gated strategy is used.

Several limitations of our study warrant mention. First, we assumed that respiratory patterns during the treatment planning CT and actual treatment were regular and identical. PCR parameters (dose rate for respective iso-layers, sweeper speed, spot position/trajectory *etc*.) are calculated from the gating window time and are sent to the irradiation machine before the delivery of each layer. Therefore, once the irradiation is started, the irradiation pattern cannot be changed even though the patient’s respiratory pattern may change. The implementation of countermeasures to irregular respiratory patterns was beyond the scope of the present study, but will be evaluated in future studies.

Second, like most simulation studies using 4DCT data, we assumed that zero motion between the respective phases of the 4DCT. That is, all calculations assumed the tumor is static within each phase and then instantaneously moves to the new position in the subsequent phase. Although with two-dimensional phantom study, we have previously confirmed its continuity [2].

Third, the respiratory phase that is determined with a non-invasive surrogate marker is not always consistent with the movement of the in-treatment tumor location. In the ongoing development of this treatment protocol, the use of fluoroscopy might allow more precise monitoring of the tumor position during treatment and it might also allow “amplitude-based gated irradiation” [14]. The evaluation of possible interplay effects with this new gating strategy must be carried out and will be addressed in a future study.

## Conclusions

PBS-CIRT with four or more PCR should substantially improve the accuracy of dose delivery for lung tumors, and bring it close to that of the planning dose distribution. In addition the use of gating reduces the residual motion and also reduces the FTV size leading to better sparring of healthy tissue while keeping the same tumor coverage. For practical treatment considerations, we do not presently calculate 4D dose distributions that include the interplay effect in our routine treatment workflow because of the costs imposed by the substantial data volume and computing time. Moreover, no commercial treatment planning system provides this type of dose calculation. The planning dose approach can therefore be integrated into routine treatment protocols in place of a complete 4D dose calculation provided that the shown approach of gating, PCR (at least four times) and if possible the usage of multiple beam ports is conducted. This simulation study provides valuable information prior to the start of PBS-CIRT in patients suffering from lung tumors.

## Abbreviations

PCR: Phase-controlled rescanning
4DCT: 4-dimensional computed tomography
CTV: Clinical target volume
OAR: Organs at risk
FTV: Field-specific target volume
D95: Dose delivered to 95% of the volume
lung-V20: Percentage of lung receiving at least 20 Gy(RBE)
PBS-CIRT: Pencil-beam scanning carbon-ion radiotherapy
NSCLC: Non-small cell lung cancer
GTV: Gross tumor volume
DIR: Deformable image registration
WEPL: Water-equivalent path length
DVH: Dose-volume histogram
Dmax: Maximum dose
Dmin: Minimum dose
HI: Homogeneity index
SD: Standard deviation
